# Optimizing ligand-receptor binding thermodynamics and kinetics: The role of Terahertz wave modulation in molecular recognition

**DOI:** 10.1016/j.fmre.2024.09.006

**Published:** 2024-09-28

**Authors:** Yibo Wang, Cong Zhang, Mingqi Li, Xiaohui Wang

**Affiliations:** a Laboratory of Chemical Biology, Changchun Institute of Applied Chemistry, Chinese Academy of Sciences, Changchun 130022, China; b School of Applied Chemistry and Engineering, University of Science and Technology of China, Hefei 230026, China

**Keywords:** Molecular recognition, Thermodynamics, Kinetics, Terahertz wave, Terahertz optogenetics

## Abstract

Precision control over ligand-receptor recognition is critical in biochemistry and pharmacology. Traditional methods that alter reaction environments have limitations in fine-tuning the thermodynamic and kinetic aspects of biochemical reactions within biological systems. The advent of terahertz wave technology represents a significant breakthrough, providing a refined approach to modulating ligand-receptor interactions. This perspective explores the cutting-edge potential of terahertz waves in refining ligand-receptor recognition, featuring their innovative application in modulating neuronal functions. The capabilities of terahertz technology to selectively influence molecular interactions are discussed, highlighting its transformative potential for advancing therapeutic strategies and deepening our understanding of biological mechanisms.

Molecular recognition, the process by which molecules interact with high specificity, is a cornerstone of biological function and drug efficacy. In biochemistry and pharmacology, understanding and manipulating ligand-receptor interactions is fundamental. These interactions are governed by the principles of thermodynamics and kinetics. Thermodynamics determines the equilibrium state of ligand-receptor interactions, affecting binding affinity and specificity through free energy changes. Kinetics, on the other hand, describes the rates at which these interactions occur, including association and dissociation rates. Traditional approaches to modulating ligand-receptor interactions rely on altering reaction conditions, including pH, ion concentration, temperature, and pressure. Alterations in pH, for instance, can affect the protonation states of specific amino acid residues in G protein-coupled receptors (GPCRs), thereby influencing both the kinetics and thermodynamics of ligand binding [1], a critical step in signal transduction pathways. Similarly, changes in ion strength can modify electrostatic interactions, impacting the binding affinity of charged ligands to their receptors. In certain cases, specific ions act as allosteric modulators, directly influencing ligand-receptor recognition in GPCRs [2]. Moreover, adjustments in temperature and pressure can affect the probability of collisions between ligands and receptors, thereby influencing ligand-protein recognition. While these methods are useful for significantly altering the thermodynamics and kinetics of ligand-receptor interactions *in vitro*, their application at the cellular or organismal level is often hindered by the potential for adverse effects or the inability to precisely control these conditions without compromising cell viability. In light of these limitations, some strategies use competing small molecules or antibodies to block or shield binding sites, offering an alternative to modulate complex biological processes without significantly altering physical conditions. However, these methods primarily affect interaction kinetics without significantly modifying thermodynamics. Meanwhile, small molecules and antibodies may lack specificity, potentially interacting with multiple targets and causing off-target effects, especially when used at high concentrations or over prolonged periods.

The interaction between electromagnetic waves and biological systems represents a cutting-edge intersection of physical chemistry and biology. Within the electromagnetic spectrum, the generalized terahertz electromagnetic waves, which range from 0.5 to 100 terahertz (equivalent to wavelengths of 3 µm to 600 µm), remain among the least explored and utilized [3]. Within this frequency range, phenomena such as the skeletal vibration of biomacromolecules and the energy absorption during rotational and vibrational transitions of dipoles in specific chemical bonds occur. When molecules are exposed to electromagnetic radiation at frequencies matching their vibrational modes, they absorb energy, which increases the amplitude of their vibrations. Therefore, terahertz waves can selectively interact with biomolecules, altering protein conformations and potentially modulating ligand-receptor binding affinities and specificities [4]. The ability of terahertz waves to penetrate the skull without damaging tissues, combined with their frequency covering the vibrational frequencies of specific chemical bonds in biomolecules, provides a new non-invasive method for probing and manipulating ligand-receptor interactions [5]. This feature makes terahertz waves an extremely attractive research tool, especially in studying the molecular recognition processes of both endogenous and exogenous ligands with their receptors. By targeting and modulating the vibrational modes of specific amino acid residues at receptor binding sites, terahertz waves can enhance binding specificity, reduce off-target effects, and potentially revolutionize the manipulation of thermodynamics and kinetics in biological systems. This approach holds significant promise for therapeutic applications and improving our understanding of fundamental biological processes.

In drug therapy, the dual challenge of enhancing efficacy while reducing toxicity remains a critical issue, with limited effective solutions currently available. Terahertz technology emerges as a potential game-changer in this context. Target proteins or key amino acid residues critical for drug binding possess unique structures and environments, which correspond to highly specific vibrational modes. By harnessing specific terahertz frequencies, molecular recognition can be enhanced by significantly strengthening the interactions between drug ligands and their designated target receptors. This can potentially increase the association rate and decrease the disassociation rate, leading to prolonged drug duration. Moreover, this approach could significantly reduce the required drug dosage while minimizing interactions with receptors linked to adverse effects, thereby decreasing unwanted side effects. This innovative approach offers a promising pathway toward refining drug therapy, aiming for maximal efficacy with minimal toxicity. Recent studies highlight the potential of psychedelics in treating neuropsychiatric disorders, including depression, anxiety, and post-traumatic stress disorder [6,7]. Nonetheless, the pronounced hallucinogenic effects and the polypharmacology of these compounds limit their clinical application. For instance, compounds such as lysergic acid diethylamide (LSD) and psilocybin interact with receptors in the central nervous system, like 5-HT_6_R and 5-HT_1A_R, facilitating therapeutic effects such as antidepressant and anxiolytic benefits [8,9]. However, their interaction with the 5-HT_2A_R receptor is associated with hallucinogenic experiences. The wide-ranging interaction with 5-HTRs leads to therapeutic gains overshadowed by significant hallucinogenic side effects. By integrating terahertz wave modulation with psychedelic therapy, it may potentially overcome these limitations. Terahertz waves could selectively enhance the interactions of psychedelics with therapeutic receptors (e.g., 5-HT_6_R) while reducing their affinity for receptors associated with hallucinogenic side effects (e.g., 5-HT_2A_R) (Fig. 1). This approach has the potential to increase the clinical adoption of psychedelics, offering therapeutic benefits while minimizing side effects. Additionally, terahertz waves offer non-invasive, real-time monitoring and dynamic modulation of biological processes, reducing the risks of tissue damage and enabling precise control over frequency, intensity, and duration to optimize therapeutic outcomes. This dynamic control allows for fine-tuning of biological responses, optimizing therapeutic outcomes, and reducing the need for repeated interventions.

**Fig. 1:**
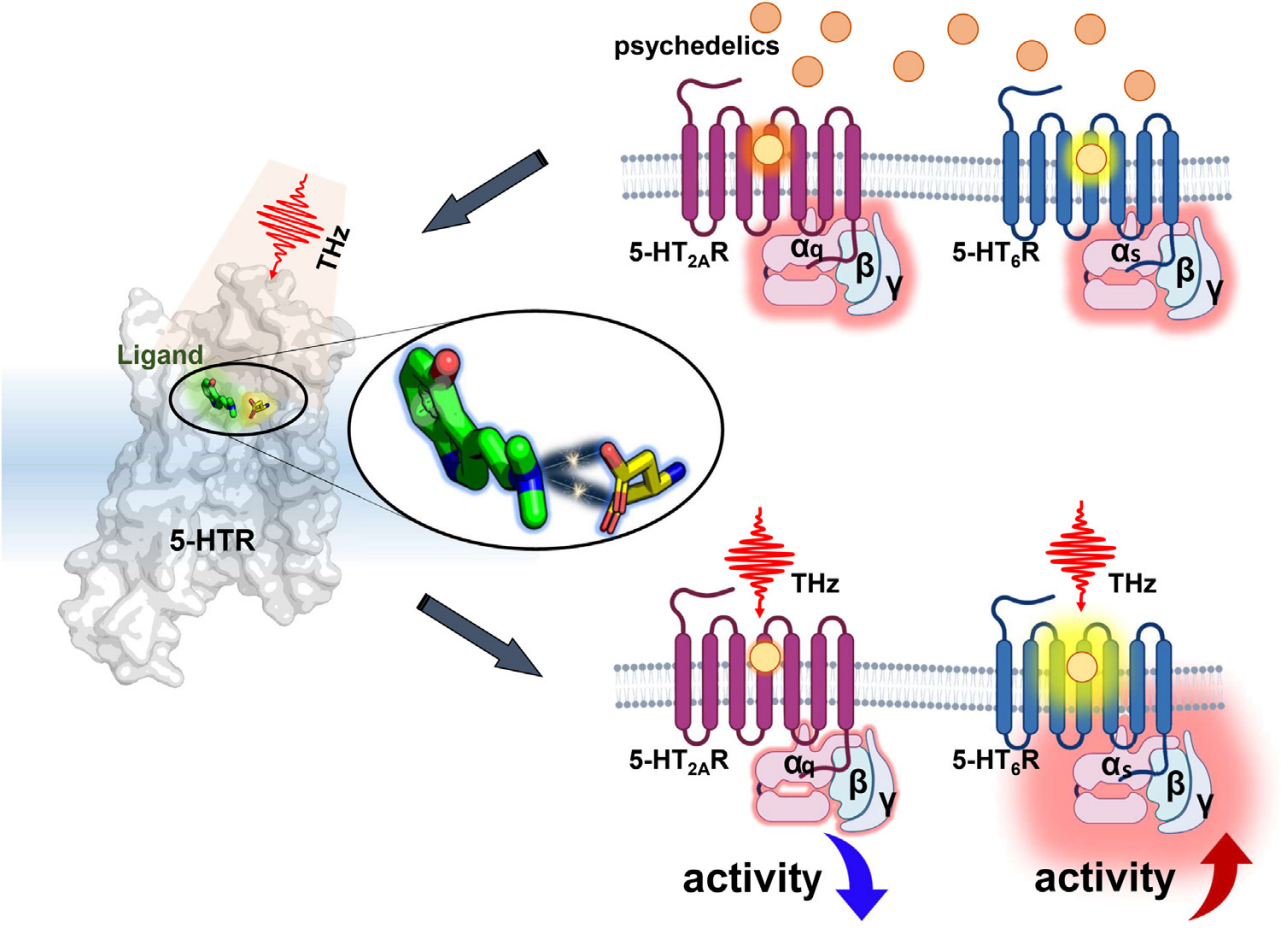
**By applying specific frequencies of terahertz waves, the targeting of psychedelics to the 5-HT receptor family can be improved without altering the structure of these substances: reducing their interaction with hallucination-related receptors (like 5-HT**_**2A**_**R), while preserving or enhancing their affinity for therapeutic-related receptors (such as 5-HT**_**6**_**R)**.

Terahertz waves can modulate the conformational flexibility of receptors, making them more amenable to ligand binding. This modulation not only improves the binding affinity but also facilitates the recognition process by ensuring that the receptor is in the optimal state for ligand interaction. Consequently, terahertz waves could become a unique tool in basic science, such as in terahertz optogenetics (Fig. 2). This approach combines the precision of optogenetics with the deep penetration capabilities of terahertz radiation to modulate neural activity non-invasively. While traditional optogenetics employs visible light, delivered through surgically implanted optical fibers, to control genetically modified neurons expressing light-sensitive proteins (opsins) [10], terahertz optogenetics explores the use of terahertz frequencies to control the opening or closing of ion channel receptors (THz opsins) without the need for invasive surgical procedures. This innovative approach promises several advantages, including the ability to reach deep brain structures without surgical intervention and minimize tissue damage due to the non-ionizing nature of terahertz radiation. However, the development of terahertz optogenetics faces challenges such as identifying or engineering opsins responsive to terahertz frequencies and creating suitable terahertz radiation sources for in vivo use. Despite these hurdles, terahertz optogenetics holds significant promise for advancing our understanding of brain functions and developing new therapeutic strategies for neurological disorders, marking a pioneering step in the convergence of biophotonics and neuroscience.

**Fig. 2:**
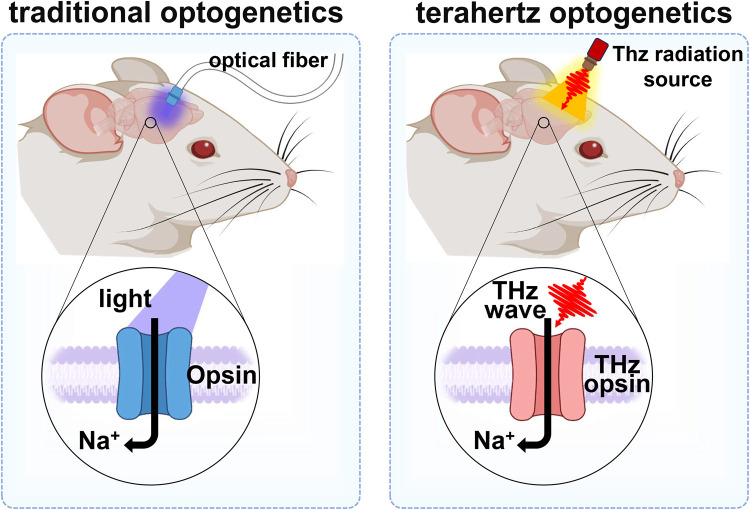
**Schematic diagram of traditional optogenetics and terahertz optogenetics**.

While the potential of terahertz modulation in ligand-receptor recognition is promising, several challenges must be addressed. One key limitation is the selective sensitivity of terahertz waves. The vibrational frequencies of receptor key residues and their characteristic functional groups fall within a relatively narrow range, influenced by their surrounding microenvironment. This can reduce the selectivity of terahertz modulation for specific target receptors. A potential solution is to incorporate unique, terahertz-sensitive functional groups into exogenous ligands that are absent in endogenous molecules. By doing so, these ligands could be selectively modulated at their characteristic terahertz frequencies, enhancing specificity. Another significant challenge lies in the absorption of terahertz waves by water, which constitutes a major component of biological tissues. Water's strong absorption properties drastically reduce the penetration depth of terahertz radiation in biological samples, complicating *in vivo* applications. To overcome this, researchers must identify terahertz frequencies that fall within the “terahertz water window”, where water absorption is comparatively lower, enabling deeper penetration while still maintaining biological relevance. Moreover, although terahertz radiation is non-ionizing and generally considered safe, the biological effects of prolonged exposure are not yet fully understood. More research is needed to explore potential long-term effects and to ensure safety before wide-scale implementation of terahertz modulation in biological systems. Despite these limitations, continued advancements in terahertz technology and methodology hold the potential to fully realize the benefits of this novel approach in molecular recognition and biochemical modulation.

In summary, terahertz radiation provides an opportunity for the selective modulation of ligand-receptor interactions. Through molecular, cellular, and animal behavioral experiments, the efficacy of this modulation can be rigorously validated. Terahertz waves provide an innovative tool for precisely controlling biochemical pathways, offering new insights into the dynamic interactions between ligands and receptors. These advancements are poised to expand our understanding of the biological effects of electromagnetic radiation and may fundamentally reshape therapeutic strategies for treating diseases.

## Declaration of competing interest

The authors declare that they have no conflicts of interest in this work.
